# Progress and challenges in sorghum biotechnology, a multipurpose feedstock for the bioeconomy

**DOI:** 10.1093/jxb/erab450

**Published:** 2021-10-13

**Authors:** Tallyta N Silva, Jason B Thomas, Jeff Dahlberg, Seung Y Rhee, Jenny C Mortimer

**Affiliations:** 1 Joint BioEnergy Institute, Emeryville, CA, USA; 2 Environmental Genomics and Systems Biology Division, Lawrence Berkeley National Laboratory, Berkeley, CA, USA; 3 Carnegie Institution for Science, Department of Plant Biology, Stanford, CA, USA; 4 UC-ANR-KARE, 9240 S. Riverbend Ave, Parlier, CA, USA; 5 School of Agriculture, Food and Wine, Waite Research Institute, University of Adelaide, SA, Australia; 6 MPI of Molecular Plant Physiology, Germany

**Keywords:** *Agrobacterium*, biofuels, bioinformatic resources, genetic engineering, genetic resources, sorghum transformation

## Abstract

Sorghum [*Sorghum bicolor* (L.) Moench] is the fifth most important cereal crop globally by harvested area and production. Its drought and heat tolerance allow high yields with minimal input. It is a promising biomass crop for the production of biofuels and bioproducts. In addition, as an annual diploid with a relatively small genome compared with other C_4_ grasses, and excellent germplasm diversity, sorghum is an excellent research species for other C_4_ crops such as maize. As a result, an increasing number of researchers are looking to test the transferability of findings from other organisms such as *Arabidopsis thaliana* and *Brachypodium distachyon* to sorghum, as well as to engineer new biomass sorghum varieties. Here, we provide an overview of sorghum as a multipurpose feedstock crop which can support the growing bioeconomy, and as a monocot research model system. We review what makes sorghum such a successful crop and identify some key traits for future improvement. We assess recent progress in sorghum transformation and highlight how transformation limitations still restrict its widespread adoption. Finally, we summarize available sorghum genetic, genomic, and bioinformatics resources. This review is intended for researchers new to sorghum research, as well as those wishing to include non-food and forage applications in their research.

## Teaser

Sorghum is a multipurpose crop resilient to suboptimal growth conditions. We highlight what genetic, genomic, and biotechnological resources are available for sorghum, with an emphasis on transformation technologies.

## Introduction

Sorghum [*Sorghum bicolor* (L.) Moench] is the world’s fifth largest cereal crop by acreage and production (FAOSTAT, https://www.fao.org/faostat/en/#data). It is an important staple food in the semi-arid tropics of Asia and Africa. Globally, sorghum is used for animal feed, fodder, and high-value products such as syrup and bioethanol. Harboring traits such as tolerance to drought, waterlogging, and salinity make it a highly productive crop in environmental conditions that restrict the cultivation of other cereals (Hadebe *et al.*, 2017; Huang, 2018). Sorghum has also been the source of exciting advances in fundamental biology such as the discovery of a metabolon for dhurrin biosynthesis (Laursen *et al.*, 2016) and a new gene and chemistry involved in conferring Striga resistance (Gobena *et al.*, 2017). Although sorghum holds great promise, it is still underutilized. In this review, we will present the current state of research employing sorghum as a multipurpose feedstock for the bioeconomy, summarize available research tools with a focus on transformation and genetic engineering, and identify promising areas for future research.

**Fig. 1.**
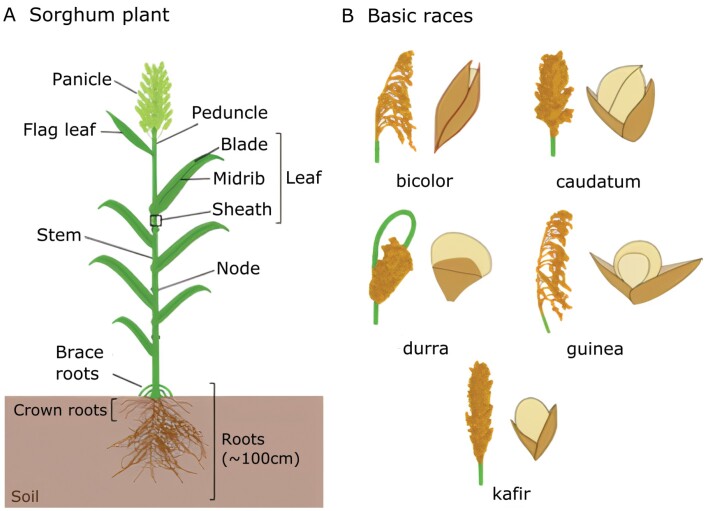
Sorghum plant morphology (A) and panicle and spikelet phenotypes of the five basic races (B). The race bicolor is the most primitive of the cultivated races and has upright semi-open panicles, with long and clasping glumes. Commercially cultivated sorghum tends to be a mixture of these major races. The race guinea originated in humid regions of West Africa and has open, elongated panicles, which helps decrease mold infection. Caudatum originated in eastern Africa and has panicles ranging from compact to open, with shorter, asymmetric, glumes that expose the grain. On the other hand, kafir, which originated in southern Africa, has tighter and longer panicles. Durras have compact panicles and originated in southern Sahara.

Cultivated sorghum (Fig. 1A) can be classified into five basic races: bicolor, guinea, caudatum, kafir, and durra, which are differentiated by the phenotype of their mature panicles and spikelets (Harlan and Wet, 1972) (Fig. 1B). *Sorghum bicolor* (L.) Moench subsp. *bicolor* contains all the cultivated sorghum varieties (Dahlberg, 2000). Sorghum can also be classified based on its agronomic characteristics into forage, biomass, sweet, and grain types (Table 1). Forage sorghum is tall, and the biomass is used to feed livestock. Important traits of forage sorghum include digestibility, nutrient content, and palatability. Biomass sorghum is bred to maximize vegetative yields, with reports of up to 61 Mg ha^–1^ (Snider *et al.*, 2012), but, unlike forage sorghum, palatability is not a concern. Some of the original biomass breeding stock was derived from forage sorghum, so high-biomass sorghums can also be produced for forage (Venuto and Kindiger, 2008). Dedicated biomass sorghum is used to produce biofuels and chemicals from the lignocellulosic biomass (cell wall), fibers for biomaterials, and biogas via anaerobic digestion (Reddy and Yang, 2005; Wannasek *et al.*, 2017; Silva and Vermerris, 2020). Sweet sorghum accumulates large amounts of soluble sugars (sucrose, glucose, and fructose) in its stems and was initially identified as an alternative sugar source in areas unsuitable for sugarcane production. Besides its use for syrup production, it can also be used for biofuel production and high-sugar forage (Rooney *et al.*, 2007). Sorghum is typically a photoperiod-sensitive plant, requiring short days (8h/16h light/dark) to transition from the vegetative to the reproductive stage. Hybrid grain sorghum is photoperiod insensitive, meaning it can flower rapidly even in the summer in temperate regions, and therefore has shorter stature and reaches maturity earlier (Smith and Frederiksen, 2000). Grain sorghum is grown for its seeds and is used as a staple food mainly in the semi-arid tropics of Asia and Africa, as animal and poultry feed, as well as a sugar source for distillation into alcohol. Recently, grain sorghum has become more popular in other countries because of its health benefits, such as reducing rates of cardiovascular disease, obesity, and certain types of cancer (reviewed in Awika and Rooney, 2004). Certain genotypes contain 3–4 times more anthocyanin, a plant pigment which has antioxidant properties, compared with other grains (Awika *et al.*, 2004). It is also a gluten-free alternative for people with celiac disease. However, in countries such as the USA, grain sorghum is primarily used to feed livestock and produce pet food, with approximately one-third of its production being directed to produce biofuels (United Sorghum Checkoff Program, https://www.sorghumcheckoff.com/).

**Table 1.** Characteristics of sorghum groups

	Forage	Sweet	Grain	Biomass
Height (m)	1.8–3.6	>3	0.6–1.2	3.5–6
Traits	Single or multicut harvest, digestibility, nutrient content, palatability	Large amount of soluble sugars in stems	Photoperiod sensitive and insensitive, high grain yield	Photoperiod sensitive, dual-purpose, high lignocellulosic biomass
Uses	Livestock feed	Syrup and biofuel production, high-sugar forage	Seed as staple food in some regions, livestock feed and biofuel production	Biofuel, biogas, and biomaterial production

Independent of the usage type, sorghum is an attractive crop for cultivation in a wide range of environments (tropical, subtropical, and temperate regions) and in soils that are considered marginal for other food crops such as maize (Fu *et al.*, 2016; Ameen *et al.*, 2017). Sorghum can grow in mineral-rich soils with pH values that limit profitable cultivation of other crops (Smith and Frederiksen, 2000). It also requires less water and exhibits drought and waterlogging tolerance (Rosenow *et al.*, 1983; Promkhambu *et al.*, 2010; Varoquaux *et al.*, 2019). As an adaptive mechanism, sorghum becomes dormant during severe drought conditions and resumes growth when re-exposed to water (reviewed by Assefa *et al.*, 2010). Another post-flowering drought adaptation is known as non-senescence or stay-green (Borrell *et al.*, 1999, 2000; Borrell and Hammer, 2000). The stay-green trait allows delayed remobilization of nitrogen in the leaves, maintaining photosynthetic activity and carbohydrate supply to the developing grain, which results in higher biomass and grain yield (Borrell *et al.*, 1999, 2000; Borrell and Hammer, 2000). For instance, stay-green sorghum hybrids can produce 47% more post-anthesis biomass under drought conditions (Borrell *et al.*, 2000). Along with drought tolerance, sorghum is also heat tolerant (Craufurd and Peacock, 1993; Nguyen *et al.*, 2013), which is particularly relevant, considering climate change predictions that include reductions in rainfall and increases in temperature in many cereal-growing regions.

## Sorghum as a multipurpose feedstock for the bioeconomy

While sorghum is an important staple food and forage crop globally, it has potential as a feedstock for renewable fuel and bioproducts (U.S. Department of Energy, 2016, https://www.energy.gov/eere/bioenergy/2016-billion-ton-report). For it to be a viable feedstock, agronomic and biomass compositional traits will likely need to be further developed to make the economics of the manufacturing processes comparable with those for fossil fuel-derived products (Baral *et al.*, 2019; Yang *et al.*, 2020, 2021). This is exemplified by the US Department of Energy Bioenergy Research Centers, which have been funded since 2007 to investigate all aspects of advanced biofuel production process (https://genomicscience.energy.gov/centers/). Sorghum is one of three DOE flagship biomass crops, and open research questions include biomass improvement and co-production of valuable chemicals. Sorghum’s versatility in multiple processing configurations is one of its key appeals (Stamenković *et al.*, 2020). For example, biodiesel can be produced from sorghum grains after pressing and transesterifying lipids (Ved and Padam, 2013). Starch from the grain or the sucrose-rich juice from the stems of sweet sorghum can be used for fermentation into biofuels and bioproducts. Beyond this, sorghum, especially the photoperiod-sensitive varieties, can produce large amounts of aerial lignocellulosic biomass that can also be used as a sustainable and economically feasible feedstock for conversion. Because of sorghum’s versatility, designing an ideal sorghum ideotype is challenging (Yang *et al.*, 2021). Instead, it is more likely that a range of sorghum varieties will continue to be developed, with their phenotype tuned to the desired downstream market.

### Target traits for biomass improvement: the cell wall

The cell wall is a crucial organelle for cell structure and protection, and is made up mainly of cellulose, hemicelluloses, and lignin. Cellulose, which constitutes 25–35% of the sorghum biomass, is made of β-1,4-glucose chains, which in turn form crystalline fibrils via hydrogen bonding (Ioelovich, 2008; Polko and Kieber, 2019). Hemicelluloses are a collection of branched hetero-polysaccharides (Ebringerová *et al.*, 2005), but in the sorghum cell wall, glucuronoarabinoxylans dominate, making up ~35% of the total biomass (Anglani, 1998; Xu *et al.*, 2018). Lignins are complex branched polyphenolics, made up of monolignol subunits derived from phenylalanine and tyrosine metabolism, and are found only in some secondary cell walls (Boerjan *et al.*, 2003). Sorghum lignin content varies between ~2% and 11% of dry matter depending on the cultivar (Brenton *et al.*, 2016), and is a key factor affecting forage palatability and biorefinery efficiency.

During biomass processing in a biorefinery, cellulose, hemicelluloses, and soluble sugars can be converted to monosaccharides, which can then be utilized as a carbon source by microbes. Most microbes preferentially use hexose sugars (such as the glucose in cellulose) over pentose sugars (such as the xylose and arabinose in xylan), so biomass with a high hexose:pentose ratio, namely reduced xylan, is preferable (Brandon *et al.*, 2020). Branched hemicelluloses such as xylan require multiple enzymes to hydrolyze them to monosaccharides, so hemicelluloses with fewer branches, or altered branch frequency, may also be preferable (Gao *et al.*, 2020). However, the cell wall should not be weakened so much that the plant lodges in the field or is more susceptible to pathogens and pests. Susceptibility to lodging and diseases due to cell wall modifications have proved difficult to predict, with some plants with engineered walls being more resistant to pathogens (reviewed by Miedes *et al.*, 2014).

In addition to sugar engineering, lignin can be modified for biomass improvement. An ideal biomass feedstock would have low lignin, since it both physically shields polysaccharides from polysaccharide-degrading enzymes and reduces enzymatic efficiency via non-specific binding. With the advent of designer lignins and use of microbes that can consume phenolics as a carbon source, monolignols are increasingly considered high-value intermediates for the production of important biochemicals (Eudes *et al.*, 2014; Karlen *et al.*, 2016; Baral *et al.*, 2019). Therefore, the desired biomass phenotypes (the sorghum biomass ideotype) will vary depending on the final target product. As a plant breeding problem, this variability highlights the need for seed producers to be able to respond rapidly to needs in the supply chain beyond their direct market (farmers), as the bioeconomy develops.

The isolation of naturally occurring lignin mutants has already proved beneficial for commercial sorghum cultivars. Nineteen *Brown midrib* (*Bmr*) mutant loci have been identified in sorghum, though only 3–4 loci are considered of agronomic interest due to their lower lignin content and higher potential for biomass conversion (Porter *et al.*, 1978; da Silva *et al.*, 2018). Engineering approaches that re-route the lignin biosynthetic pathway have been demonstrated in a number of plant species (Fu *et al.*, 2011; Eudes *et al.*, 2014; Wilkerson *et al.*, 2014; Yan *et al.*, 2018). Restricting engineering to specific cell types has been successful in reducing lignin while avoiding stem weakness (Yan *et al.*, 2018).

### Beyond biomass: oils, bioproducts, and novel materials

In addition to being a source of starch and lignocellulose to produce biofuels, sorghum has the potential to function as a factory for other bioproducts or their precursors, and this will be important for the economic success of advanced biofuels. Compared with microbial production systems, *in planta* production of chemical compounds can reduce inputs, costs of post-production conversion steps, and the amount of pathway engineering needed (Yang *et al.*, 2020). Proposed examples include pharmaceuticals (artemisinin and cannabidiol), materials (e.g. latex), insecticides (e.g. limonene), and plastic precursors [e.g. polyhydroxybutyrate (PHB)]. Modeling has shown that the added value of bioproducts can lower biofuel production costs to prices competitive with fossil fuels, as well as providing a better farmgate price for growers (Yang *et al.*, 2020). Another promising route for sorghum metabolic engineering is to target triacylglycerol (TAG) accumulation in leaves for oil production, which can be used for biodiesel production. Although vegetative organs represent most of the above-ground biomass, leaves accumulate <1% lipids (Yang and Ohlrogge, 2009), so plant oil production relies on seeds rich in TAG. However, up to an 8.4% increase of TAG in leaf tissues has been achieved in sorghum by simultaneous overexpression of the genes encoding the maize transcription factor WRINKLED1, *Umbelopsis ramanniana* acyltransferase UrDGAT2a, and *Sesamum indicum* oil body protein OLEOSIN-L, providing a basis for further improvements in levels of extractable oil for commercial purposes (Vanhercke *et al.*, 2019).

Lignin valorization is another attractive option to add value to compounds from waste products in a biorefinery (Mottiar *et al.*, 2016). Potential high-value applications of lignin range from synthesis of lignin nanotubes for gene delivery (Ten *et al.*, 2014) to development of lignin-based antibacterial products for pharmaceutical and biomedical industries, demonstrating the wide range of properties that can be exploited (Grossman *et al.*, 2020). Lignin precursors have been re-routed in tobacco to produce intermediates that can be converted by an engineered microbial chassis to produce high-value compounds pyrogallol and *cis,cis*-muconic acid (Wu *et al.*, 2017). Similar approaches could be applied to re-route higher levels of valuable intermediates in sorghum, although it will require better understanding of the regulation of cell wall biosynthesis pathways. Lignin valorization into phenolic compounds such as eugenol is also of great interest. Eugenol can be used in food, cosmetics, and pharmaceutical industries, and its high demand can lead to high market value (Martinez-Hernandez *et al.*, 2019). Techno-economic analysis (TEA) and life cycle assessments (LCA) have shown that lignin valorization into eugenol and other methoxyphenols can reduce the cost of ethanol production by up to 23% and reduce greenhouse gas emissions by up to 78% compared with the petrochemical industry (Martinez-Hernandez *et al.*, 2019). As demonstrated by this and other examples (Yang *et al.*, 2020), TEA and LCA are important resources to guide decisions on which compounds should be targeted for genetic engineering, based on their economic value.

Finally, novel materials can be produced from biomass. For example, cellulose derived from lignocellulosic material can be broken into nanofibers, which have nanostructure favorable to high mechanical performance of nanofiber networks and composite materials (Sehaqui *et al.*, 2010). Cellulose nanofibers are a great renewable material for the manufacturing of ultrafiltration membranes and can also be used as barrier layers in packaging material, among other useful applications (Forde *et al.*, 2016). Additionally, both hemicelluloses and pectins have been suggested for use in a range of materials which include medical devices (Zheng *et al.*, 2020), superconductors (Di Giacomo *et al.*, 2015), and biodegradable packaging (Gouveia *et al.*, 2019; Mendes *et al.*, 2020). Collaborations between sorghum researchers and material scientists to develop new uses for biomass components or to engineer improvements are likely to be fruitful.

## Barriers to using sorghum in biotechnology applications

There are three major barriers to the use of engineered sorghum: technical challenges around sorghum transformation, general societal concerns about engineered crops, and specific concerns about sorghum gene flow to weedy relatives. We will not dwell on the GMO issue here because it is reviewed in depth in the literature.(McHughen and Wager, 2010; National Academies of Sciences, Engineering, and Medicine, 2016; Wolt, 2017; Callaway, 2018; Spicer and Molnar, 2018; Waltz, 2018; Zhang *et al.*, 2020).

Though we describe many examples of existing transgenic sorghum technology, to our knowledge, there is no transgenic sorghum grown commercially. One main reason for the limited use of transgenic sorghum in the USA is concerns about gene flow to its sexually compatible wild weedy relatives such as Johnsongrass (*S. halepense*), *S. bicolor* subsp. *drummondii*, and *S. bicolor* subsp. *verticilliflorum* via pollen dispersal and subsequent cross-pollination and hybridization. These wild relatives can easily hybridize with the cultivated sorghum to produce the noxious weed shattercane (Ejeta and Grenier, 2005). Strategies to limit gene flow, such as male sterility, could be implemented, as could agronomic strategies which monitor for compatible weedy species within the range of pollen flow. For example, in sorghum, it has been estimated that after 700 m, very little, if any, outcrossing would be expected (Schmidt and Bothma, 2006). It is also important to note that most of the discussed modifications would likely be considered ‘null’; that is, they would not be expected to give weedy relatives a selective advantage. This makes regulation more straightforward than traits such as herbicide tolerance.

The rapid development of transgenic sorghum varieties will be necessary to complement gains from traditional sorghum breeding, as humanity faces increasing challenges from climate change, degraded soils, and increased population. In the next section, we will give an overview of the transformation methods adopted for sorghum biotechnology thus far and discuss the main bottlenecks that need to be addressed to have efficiencies comparable with other grasses and move the field forward.

## Sorghum transformation

The limited ability to transform sorghum is the major barrier to the widespread adoption of sorghum as a research model and as feedstock for the growing bioeconomy. Sorghum transformation is technically challenging, comparatively costly and time-consuming, and limited to a few genotypes. Sorghum is highly recalcitrant to tissue culture and transformation, mainly because of genotype-dependent responses, production of phenolic compounds, short-term plant regeneration ability, and acclimatization issues (the ability of plants to survive the transfer from *in vitro* culture to soil) (Maheswari *et al.*, 2006; Altpeter *et al.*, 2016). Here, we describe the achievements so far, and outline research questions that would help resolve existing barriers to sorghum engineering.

**Fig. 2.**
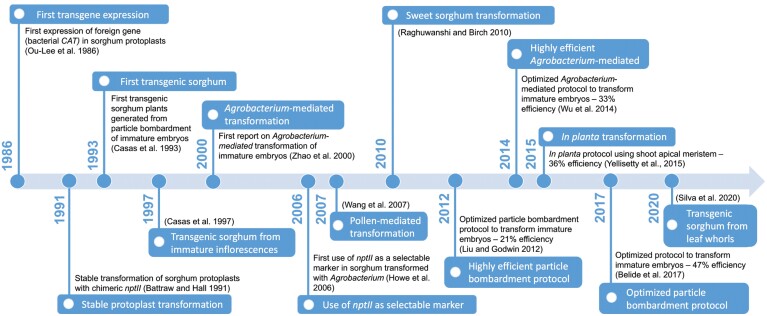
Timeline of advances in sorghum transformation. *Cat*, *chloramphenicol acetyltransferase*; *NptII*, *neomycin phosphotransferase II.*

Since transgenic sorghum was first described (Casas *et al.*, 1993), many improvements have been reported (Fig. 2). Casas and colleagues used immature embryos from the genotype P898012 to induce callus formation for particle bombardment, and obtained a transformation efficiency of 0.3% (Casas *et al.*, 1993). Since then, the process has been improved using the genotype Tx430, and reached efficiencies of up to 46.6% (Belide *et al.*, 2017). Using *Agrobacterium tumefaciens* to introduce the transgene via infection, transformation efficiency has increased from 9.7% in the initial studies (Zhao *et al.*, 2000) to 33.2% (Wu *et al.*, 2014). An important factor for tissue culture and, consequently, transformation success, is genotype selection. For the past 10 years, the grain sorghum inbred line Tx430 has been routinely used due to its consistently high callus induction and regeneration frequencies (Howe *et al.*, 2006; Gurel *et al.*, 2009; Liu and Godwin, 2012; Wu *et al.*, 2014; Liu *et al.*, 2015; Belide *et al.*, 2017). However, Tx430 was directly compared with seven bioenergy parental sorghum lines using the protocols from Liu and Godwin (2012) and Wu *et al.* (2014). While Tx430 had high callus proliferation accompanied by low phenolic release, lines PI329311 and Rio had the best regeneration rates (Flinn *et al.*, 2020).

The explant source also plays a role in transformation efficiency. A variety of explants, such as immature and mature embryos, immature inflorescences, leaf discs, leaf whorls, and shoot meristems, have been used (Tables 2, 3). The most successful studies have used immature embryos due to their high embryogenic and regeneration competence (Tables 2, 3). However, the plant needs to reach the reproductive stage, which is limited to specific seasons or periods of time, and the narrow time window of 10–15 d in which the immature seeds need to be collected. To overcome these drawbacks, Silva *et al.* (2020) tested leaf whorls from the genotypes Tx430 and P898012, since this material can be collected throughout the year. The protocol also saves at least 4 weeks, as the explants can be collected around 30 d after emergence, compared with 70 d needed to collect immature embryos. Furthermore, the excision of leaf whorls is more technically straightforward than embryo isolation, allowing higher throughput.

**Table 2.** Relevant literature regarding sorghum transformations and their use in research articles

References	Explant	Citations^a^	Use in research article methods (References)
Electroporation			
Ou-Lee *et al.*, (1986)	Protoplasts	105	0
Battraw and Hall, (1991)	Protoplasts	36	0
Pollen sonication			
Wang *et al.*, (2007)	Pollen	17	0
Particle bombardment			
Casas *et al.*, (1993)	Immature embryos	125	5 (Casas *et al.*, 1997; Emani *et al.*, 2002; Jeoung *et al.*, 2002; Grootboom *et al.*, 2008; Kumar *et al.*, 2011)
Casas *et al.*, (1997)	Immature inflorescences	43	1 (Sato *et al.*, 2004)
Able *et al.*, (2001)	Immature embryos	43	0
Tadesse *et al.*, (2003)	Immature embryos	49	2 (Grootboom *et al.*, 2008; Brandão *et al.*, 2012)
Grootbroom *et al.*, (2010)	Immature embryos	25^b^	1 (Grootboom *et al.*, 2014)
Raghuwanshi and Birch, (2010)	Immature embryos	28	0
Liu and Godwin, (2012)	Immature embryos	50	12 (Liu *et al.*, 2013, 2015, 2019; Cardinal *et al.*, 2016; Do *et al.*, 2016; Belide *et al.*, 2017; Lamont *et al.*, 2017; Liu *et al.*, 2017; Schnippenkoetter *et al.*, 2017; Vanhercke *et al.*, 2019; Flinn *et al.*, 2020; Silva *et al.*, 2020)
Brandão *et al.*, (2012)	Immature inflorescences	3^b^	0
Visarada *et al.*, (2014) ^c^	Immature embryos and shoot buds	10	1 (Visarada *et al.*, 2016)
Belide *et al.*, (2017)	Immature embryos	8	1 (Vanhercke *et al.*, 2019)
*Agrobacterium-*mediated transformation			
Zhao *et al.*, (2000)	Immature embryos	125	12 (Gao *et al.*, 2005; Howe *et al.*, 2006; Nguyen *et al.*, 2007; Lu *et al.*, 2009; Silva *et al.*, 2011; Lipkie *et al.*, 2013; Visarada *et al.*, 2014; Wu *et al.*, 2014; Cho *et al.*, 2014; Elkonin *et al.*, 2016; Assem *et al.*, 2017; Kuriyama *et al.*, 2019)
Carvalho *et al.*, (2004)	Immature embryos	76^b^	2 (Nguyen *et al.*, 2007; Raghuwanshi and Birch, 2010)
Gao *et al.*, (2005)	Immature embryos	67	3 (Gurel *et al.*, 2009; Liu *et al.*, 2013; Chou *et al.*, 2020)
Howe *et al.*, (2006)	Immature embryos	79	12 (Kumar *et al.*, 2011; Mall *et al.*, 2011; Kumar *et al.*, 2012; Jiang *et al.*, 2013; Dwivedi *et al.*, 2014; Elkonin *et al.*, 2016; Scully *et al.*, 2016; Peña *et al.*, 2017*a*, *b*; Cuevas *et al.*, 2016; Pan *et al.*, 2018; Kempinski *et al.*, 2019)
Nguyen *et al.*, (2007)	Immature embryos	41	0
Gurel *et al.*, (2009)	Immature embryos	71	4 (Singh *et al.*, 2012; Liu *et al.*, 2013; Hayta *et al.*, 2019; Kuriyama *et al.*, 2019)
Wu *et al.*, (2014)	Immature embryos	64	7 (Cho *et al.*, 2014; Magomere *et al.*, 2016; Yamaguchi *et al.*, 2016; Che *et al.*, 2018; Kuriyama *et al.*, 2019; Flinn *et al.*, 2020; Aregawi *et al.*, 2020, Preprint)
Yellisetty *et al.*, (2015)	Shoot apical meristem - *in planta*	5	0
Do *et al.*, (2016)	Immature embryos	18	3 (Mookkan *et al.*, 2017; Do *et al.*, 2018; Char *et al.*, 2020)

Citation count checked on 12 November 20, based on CrossRef (source indicated when CrossRef was not available).

Citation count based on Google Scholar metrics

Tested both particle bombardment and *Agrobacterium*-mediated transformation

**Table 3.** Main sorghum transformation methods, explants, genotypes, selectable markers, optimizations, and *Agrobacterium* strains, when appropriate, adopted for improvements in tissue culture and transformation efficiency

References	Explants^a^	Genotypes^a^	*Agrobacterium* strain^a^	SM^a^	Max. TE	Optimizations
Particle bombardment						
Casas *et al.*, (1993)	Immature embryos	P898012	–	*Bar*	0.29%	Genotypes (IS4225, CS3541, M91051, Tx430, P898012, P954035, SRN39, and Shanqui red)
Able *et al.*, (2001)	Immature embryos	SA281	–	*Bar*	3 out of 4 tested events	Genotypes (M35-1, SA281, QL41, and P898012), explant (immature embryos and leaf segments), promoters (*Act1*, *CaMV35S*, and *Ubi*) and biolistic parameters (acceleration pressure, distance to target tissue from expulsion point, aperture of helium inlet valve)
Tadesse *et al.*, (2003)	Immature embryos	Ethiopian accession ‘214856’	–	*Npt*	1.30%	Explants (immature and mature embryos, shoot tips, calli), promoters (*Act1D*, *Adh1*, *CaMV35S*, and *Ubi1*), selectable markers (*Bar* and *Npt*) and biolistics parameters (acceleration pressure, target distance, gap width and travel distance)
Casas *et al.*, (1997)	Immature inflorescences	SRN39	–	*Bar*	2.61%	Genotypes (M91051, P898012, P954035, PP290, and SRN39), panicle length and biolistic parameters (particle size and material, DNA amount, acceleration pressure and target distance)
Grootbroom *et al.*, (2008)	Immature embryos	P898012	–	*Pmi*	0.77%	Selectable markers (*Bar* and *Pmi*)
Raghuwanshi and Birch, (2010)	Immature embryos	Ramada	–	*Hpt*	0.09%	Genotypes (32 sweet sorghum), tissue culture media composition (increase of cytokinin), selectable markers (*Hpt* and *NptII*)
Liu and Godwin, (2012)	Immature embryos	Tx430	–	*NptII*	20.70%	Tissue culture media composition and biolistics parameters
Brandão *et al.*, (2012)	Immature inflorescences	CMSXS102B	–	*Bar*	3.33%	Genotypes (nine accessions from Embrapa Maize and Sorghum National Research Center, Brazil), explant developmental stages (3–5cm in length), biolistics parameters (in osmotic medium, acceleration pressure, microcarriers flying distance)
Visarada *et al.*, (2014)	Immature embryos	CS3541 and 296B	–	*Bar*	0.25%	Delivery method (*Agrobacterium* and particle bombardment), explant size, post-bombardment treatments
Belide *et al.*, (2017)	Immature embryos	Tx430	–	*NptII*	46.60%	Tissue culture media composition (addition of lipoic acid), explant size, selectable markers (*Bar* and *NptII*), method of subculture post-bombardment
Silva *et al.*, (2020)	Leaf whorls	Tx430	–	*NptII*	All 7 tested events	Genotypes (Tx430 and P898012) and tissue culture media composition (addition of activated charcoal and polyvinylpyrrolidone)
*Agrobacterium*-mediated transformation						
Zhao *et al.*, (2000)	Immature embryos	P898012	LBA4404	*Bar*	10.10%	Genotypes (P898012 and PHI391), source of explant (grown in the field or greenhouse), tissue culture conditions and media composition
Carvalho *et al.*, (2004)	Immature embryos	P898012	LBA4404	*Hpt*	3.50%	Genotypes (Feterita Gesish, P898012, P967083, IS2329, Rio, Sugar drip, B-Wheatland, RTx430, and Candystripe), embryo selection, tissue culture media composition, tissue culture conditions, *Agrobacterium* inoculation methods
Gao *et al.*, (2005)	Immature embryos	C401	EHA101	*Pmi*	3.30%	Genotypes (C401 and Pioneer 8505), tissue culture media composition
Howe *et al.* (2006)	Immature embryos	C2-97	NTL4	*NptII*	4.50%	Genotypes (Tx430 and C2-97), *Agrobacterium* strains (C58C1, LBA4404, EHA101, C58, and NTL4), selection agent (geneticin and paromomycin)
Nguyen *et al.*, (2007)	Immature embryos	Sensako 85/1191	LBA4404	*Hpt*	5.00%	Explant pre-treatment, tissue culture conditions and media composition
Gurel *et al.*, (2009)	Immature embryos	P898012	LBA4404	*Pmi*	8.30%	Genotypes (P898012, Tx430, 296B, and C401), explant pre-treatmemt, *Agrobacterium* strains (EHA101 and LBA4404), tissue culture media composition
Visarada *et al.*, (2014)	Immature embryos and shoot buds	CS3541 and 296B	EHA105	*Bar*	0.23%	Delivery method (*Agrobacterium* and particle bombardment), explant type (shoot buds and immature embryos), decontamination treatments for removal of *Agrobacterium*
Wu *et al.*, (2014)	Immature embryos	Tx430	AGL1	*Pmi*	33.20%	*Agrobacterium* strains (AGL1 and LBA4404), selectable markers (*PAT* and *Pmi*), tissue culture media composition (increased copper sulfate and plant hormone BAP)
Yellisetty *et al.*, (2015)	Shoot apical ­meristem	SPV462	LBA4404	*Hpt*	36%^b^	*In planta* method development
Do *et al.*, (2016)	Immature embryos	P898012	AGL1	*Bar*	14.20%	Genotypes (P898012, TBx623, Tx2737, Tx430, and Wheatland), *Agrobacterium* strains (AGL1, EHA101, and GV3101), promoters (*CaMV35S*, *MAS*, and *Ubi*), tissue culture conditions
Sato-Izawa *et al.*, (2018)	Immature embryos	Tx430	GV2260	*Hpt*	1.90%	Explant pre-treatment and size

Selectable markers (SM): *Bar*, *Bialaphos resistance*; *Hpt*, *Hygromycin phosphotransferase*; *Npt*, *Neomycin phosphotransferase*; *PAT*, *Phosphinothricin acetyltransferase*; *Pmi*, *Phosphomannose isomerase*. Promoters: Act1D, *Actin 1D*; *Adh1*, *Alcohol dehydrogenase isozyme 1*; *CaMV35S*, *Cauliflower mosaic virus 35S; MAS*, *Mannopine synthase*; *Ubi*, *Ubiquitin*. Max. TE: maximum transformation efficiency. TE is generally defined as the total number of independent events regenerated divided by the total number of transformed explants, although it can be omitted or vary depending on the publication.

Results from the most successful transformations or optimized conditions.

Results from T_1_ from selected positive T_0_ plants.

### Current transformation methods

**Fig. 3.**
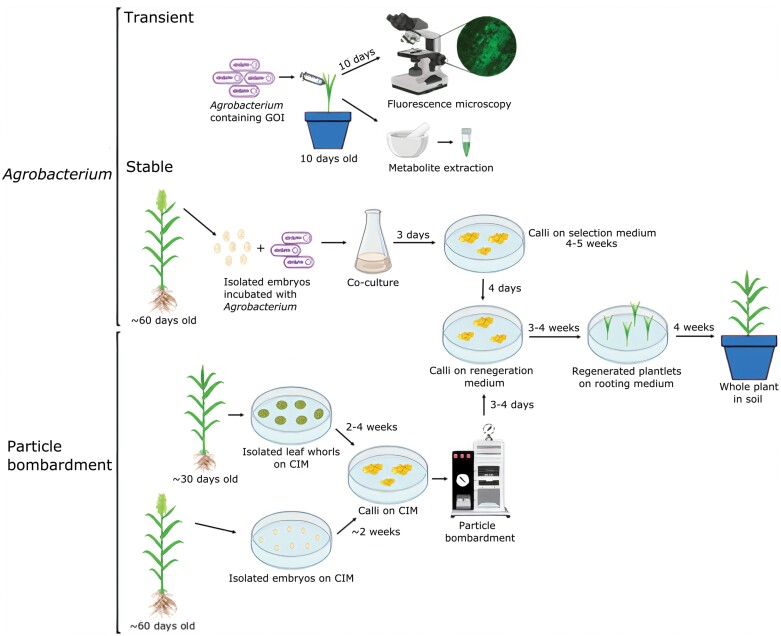
Representation of transformation methods adopted for sorghum. GOI, gene of interest; CIM, callus induction media.

To improve sorghum tissue culture and transformation, different genotypes, transformation methods, and explant sources have been tested over the years. The improvements resulted in reported increases in transformation efficiency from 0.3% to 46.6%, but these remain restricted to select genotypes, and hampered by the seasonality of explant availability. Thus far, four transformation methods have been reported for stable and transient gene expression in sorghum: electroporation; pollen-mediated transformation; particle bombardment; and the *Agrobacterium*-mediated method. Of these, particle bombardment and *Agrobacterium*-mediated transformation have been widely tested (Fig. 3). In this section, we summarize these methods, including the extent of their published usage following the initial report, which we use as a proxy for robustness, in Tables 2, 3. We will focus here on the two most commonly used methods: particle bombardment and *Agrobacterium*-mediated transformation.

#### Particle bombardment

Particle bombardment, also called biolistics or the gene gun method, physically delivers DNA into intact cells or tissues. It is based on high-speed acceleration of DNA-coated gold or tungsten particles (Sanford *et al.*, 1987). The method overcomes the host range restrictions faced when using *Agrobacterium* and viral vectors. Furthermore, since it does not introduce additional non-plant-derived DNA elements into the plant (as with *Agrobacterium*-mediated methods), it can simplify transgenic crop regulation. Particle bombardment has also been used for plastid transformation. Since plastids are maternally regulated, this can also aid control of gene flow (Svab *et al.*, 1990; Svab and Maliga, 1993; Kumar *et al.*, 2004; Dufourmantel *et al.*, 2007; Lu *et al.*, 2013; Li *et al.*, 2016). The method of Liu and Godwin (2012), currently the most widely prescribed for sorghum transformation using particle bombardment (as judged by citation count in peer-reviewed literature outlined in Table 2), obtained a transformation efficiency of 20.7% with an optimized protocol using Tx430 immature embryos. Moreover, >90% of the transgenic plants exhibited normal growth and fertility. Adding lipoic acid to the medium and splitting the calli further enhanced the callus induction rate (Belide *et al.* 2017) (Table 3).

A major drawback of particle bombardment is the random integration of multiple copies of the transgene into the genome, which can lead to transgene rearrangements and silencing (reviewed by Kohli *et al.*, 2003). However, optimization of the procedure can result in single or a low number of transgene copies (Yao *et al.*, 2006). Random integration can also be mitigated by using approaches such as genomic safe harbors: sites in the genome that accommodate transgenes without unwanted interactions (Papapetrou and Schambach, 2016). For example, (Dong *et al.*, 2020) have achieved targeted insertion of a 5.2kb carotenoid biosynthesis cassette at two pre-determined genomic safe harbors in rice. Therefore, this approach could potentially be applied to any crop species. Another issue with particle bombardment is the variable transformation efficiency among genotypes. For example, the elite parental lines CS3541 and 296B were transformed to increase stem borer resistance, but the highest transformation efficiency obtained was 0.25% (Visarada *et al.*, 2014). Traditionally, the most extensively studied genotypes belong to the grain sorghum category, so reported advances are mostly applicable to that type of sorghum. For example, a comparison of 32 sweet sorghum varieties reported a transformation efficiency maximum of 0.09% (Raghuwanshi and Birch, 2010). To fully exploit sorghum as a multipurpose crop that supports the growing bioeconomy, it will be necessary to easily transform many sorghum types, including biomass and forage varieties. Finally, a recent study showed, using whole-genome sequencing, that particle bombardment can frequently induce large-scale genome damage and rearrangement (J. Liu *et al.*, 2019). This can be problematic both for researchers, as this may impact phenotype, and potentially for regulators, as it may increase the risk of the crop being a food safety hazard. Therefore, *Agrobacterium*-mediated transformation, despite its drawbacks as discussed below, is still considered the preferred method of transformation by most.

#### *Agrobacterium*-mediated transformation

*Agrobacterium tumefaciens* mediated-transformation (reviewed by Gelvin, 2003) was initially used in eudicotyledonous plants, since monocotyledons are not natural hosts of *A. tumefaciens*. However, successful transformations of many monocots, such as barley, maize, rice, sorghum, and wheat, have now been achieved (Hiei *et al.*, 1994; Ishida *et al.*, 1996; Cheng *et al.*, 1997; Zhao *et al.*, 2000). *Agrobacterium*-mediated transformation is generally preferred when the goal is to produce plants with single- or low-copy inserts. This approach also has the advantage of resulting in minimal rearrangement of the integrated transgene.

The first reported use of *Agrobacterium* for stable sorghum transformation was from Zhao *et al.* (2000). Wu *et al.* (2014) optimized the resting and selection media by adding increased levels of copper sulfate and the plant hormone 6-benzylaminopurine (BAP) to generate high-quality, fast-growing, and regenerable transgenic calli. They also tested different *Agrobacterium* strains and selectable markers. Tx430 immature embryos infected by the *Agrobacterium* strain LBA4404 resulted in transformation efficiencies of up to 12.4% when the selectable marker adopted was *Phosphomannose isomerase* (*Pmi*), and 13.4% when *Phosphinothricin acetyltransferase* (*PAT*) was used. Using the strain AGL1 and *Pmi* selection, efficiencies of up to 33.2% were obtained, which is the most effective *Agrobacterium* protocol reported so far. The authors also point out that the size of T-DNA impacts the quality event frequency, as lower frequency was obtained when larger T-DNA was used (16.3kb versus 7.9kb). Quality events are defined as transformants with intact single copies of T-DNA integrated in the genome without the presence of a vector backbone.

Another optimization of transformation and regeneration with *Agrobacterium* was achieved by using standard binary vectors containing the *Bar* gene as the selectable marker under the control of a *Mannopine synthase* (*MAS*) promoter and the *Agrobacterium* strain AGL1 to transform immature embryos from P898012 (Do *et al.*, 2016). Activities of modified *Cauliflower mosaic virus 35S* (*CaMV35S*), maize *Ubiquitin* (*Zm-Ubi*), and *MAS* promoters were evaluated, and the highest transformation efficiency was achieved using *MAS*. Additionally, transformation efficiency was significantly improved using a standard binary vector, while studies that achieved higher efficiencies, such as that of Wu *et al.* (2014), adopted superbinary vectors. Superbinary vectors have additional virulence genes from a Ti plasmid, which is beneficial for recalcitrant plants (Komari *et al.*, 2006), but are challenging for vector construction, cloning, and transformation. The authors achieved a regeneration time frame of 7–12 weeks and an overall transformation efficiency of 14% (Do *et al.*, 2016).

### Improving *Agrobacterium* transformation efficiency with morphogenic regulators

To increase efficiency of transformation and expand the range of genotypes amenable to transformation, growth-stimulating morphogenic regulators have been used to induce somatic embryogenesis in monocots (Lowe *et al.*, 2016; Mookkan *et al.*, 2017; Nelson-Vasilchik *et al.*, 2018). Morphogenic regulators are genes involved in developmental processes that control morphogenesis such as embryo and meristem development. Lowe *et al.* (2016) successfully introduced the morphogenic regulators *Baby boom* (*Bbm*) and *Wuschel2* (*Wu2*) in maize, sorghum, and rice using the *Agrobacterium* strain LBA4404 and in sugarcane using the strain AGL1. Although morphogenic regulators promote the induction of somatic embryogenesis, they also cause calli necrosis, preventing the regeneration of transgenic plants (Lowe *et al.*, 2016). To overcome this, a CRE/lox recombination system under the control of a desiccation-induced gene (*Rab17*) was used to remove the region of the expression cassette containing *Bbm* and *Wu2*. Transgenic calli are then subjected to desiccation prior to regeneration, allowing production of healthy transgenic plants. In sorghum, using Tx430 immature embryos as the starting material, the transformation efficiency improved from 1.9% to 18.3% when *Bbm* and *Wu2* were introduced simultaneously (Lowe *et al.*, 2016).

Although morphogenic regulators represented a significant improvement, higher transformation efficiencies of 33.2% (Wu *et al.*, 2014) and 46.6% (Belide *et al.*, 2017) were obtained with the genotype Tx430 using traditional methods. The most compelling argument for the morphogenic regulator method is the possibility of transforming genotypes that are currently recalcitrant to transformation. However, to date in sorghum, this approach has been reported mostly in transformable cultivars (Lowe *et al.*, 2016; Mookkan *et al.*, 2017). Mookkan *et al.* (2017) used the *Agrobacterium* strain EHA101 to transform immature embryos from sorghum genotype P898012 with a vector containing *Bbm*, *Wu2*, and the desiccation-inducible CRE/lox recombination system. They observed that calli transformed with *Bbm* and *Wu2* reached up to 54.5% of green fluorescent protein (GFP) expression, while calli transformed with vectors without them did not show any GFP expression. Additionally, Nelson-Vasilchik *et al.* (2018) published a protocol using the genotype BTx623, besides the previously reported P898012, for *Agrobacterium*-mediated transformation with the same morphogenic regulators, the *Rab17*_pro_:CRE/lox-inducible system, and *Agrobacterium* strains AGL1 and EHA101, and showed a regeneration rate of ~15%.

### *In planta* transformation

*Agrobacterium tumefaciens* has also been used for *in planta* transformation in sorghum, which allows the introduction of DNA directly into intact plant tissue, removing the dependence on tissue culture and regeneration protocols. Yellisetty *et al.* (2015) reported an *in planta* transformation method where *Agrobacterium* strain LBA4404 was inoculated onto the shoot apical meristems of germinating sorghum seedlings, with transformation efficiencies of up to 36%. Despite these high reported efficiencies, the method has not been applied to further studies (Table 2). Haploid egg cell transformation by floral dipping is widely used for *A. thaliana* and has been applied to other Brassicaceae species, flax, and even a grass *Setaria viridis* (Liu *et al.*, 2012; Martins *et al.*, 2015). However, in planta transformation has not been established as a standard protocol for many species due to a lack of reproducibility (Hamada *et al.*, 2017). Fundamental understanding of why some plants, such as *A. thaliana* and *Camelina sativa*, are susceptible to *Agrobacterium* haploid egg cell transformation would be an important step forward in plant science, as this could lead to application of this method to other species, such as sorghum.

### Transient expression

The methods discussed above are mainly used for stable transformation, in which the genes are integrated into the host chromosomes and are inherited through subsequent generations. Stable transformation is particularly interesting if the goal is to engineer traits in the long term. However, for studies aiming at gene characterization, vector validation, or protein subcellular localization, especially in recalcitrant species such as sorghum, transient expression is a valuable and time-saving tool. It allows temporary expression of the introduced genes, which do not integrate into the host genome, but uses its transcriptional and translational machinery to synthesize the desired proteins. Transient expression generally reaches its maximum level between 18-48h after transformation and persists for a few days (Abel and Theologis, 1994). *Agrobacterium*-mediated transformation can also be successfully applied to transient expression. For example, Sharma *et al.* (2020) developed an in planta method using *Agrobacterium* for infiltration in leaves of 3- to 4-week-old sorghum, in which GFP expression was detected 3–4 d after infiltration. The method was also used to demonstrate clustered regularly interspaced short palindromic repeats (CRISPR)-mediated genome editing as a promising approach to test single-guide RNA (sgRNA) efficiencies *in vivo* (Liang *et al.*, 2019).

### Future needs for sorghum transformation

Although there has been progress, the technical challenges associated with sorghum tissue culture and transformation mean that efficiencies still lag behind most other monocot crops such as rice, which routinely reaches efficiencies of up to 90% (Hiei and Komari, 2008). To move the field forward, the main bottlenecks that need to be addressed are genotype dependence, prevention of transgene flow to wild relatives, and achieving higher transformation efficiency reproducibly. Overcoming these bottlenecks will allow the efficient application of synthetic biology principles, and the direct engineering of elite germplasm. In particular, this will enable the routine use of gene editing tools, including CRISPR/Cas systems and successful metabolic engineering for high-value traits. Here, we highlight some key areas for future research.

### Genotype independence

Currently, the inbred lines Tx430 and P898012 are the most used genotypes for transformation due to their higher embryogenic capacity. This is limiting, particularly for commercial purposes, where the engineering of elite cultivars would be beneficial. What underpins these genotypic differences in transformability is not known. However, overexpression of morphogenic regulators such as *Bbm* and *Wus2* has the potential not only to induce somatic embryogenesis in an expanded range of genotypes, but also to bypass or accelerate tissue culture via *de novo* meristem formation as demonstrated in eudicots (Maher *et al.*, 2020). Assessment of other morphogenic regulators such as *Leafy cotyledon1* (*Lec1*), *Lec2*, *Monopteros* (*MP*), *Shoot meristemless* (*STM*), hormone biosynthetic genes such as *Isopentenyl transferase* (*Ipt*), and their combinations are all promising strategies.

Besides using morphogenic regulators, genotype independence can be achieved by using tissues other than embryos, which has been achieved in barley, cotton, and rice (Dey *et al.*, 2012; Ma *et al.*, 2013; Han *et al.*, 2021). For example, Han *et al.* 2021)successfully used microspores from barley anthers to induce callus formation for transformation and CRISPR/Cas gene editing. The diverse genetic backgrounds of the tested varieties indicated that the method was genotype independent and could be expanded to other species with established anther culture protocols. Additionally, shoot apices from 3- to 5-day-old seedlings have been used for *Agrobacterium* infection of cotton and rice for development of genotype-independent regeneration protocols (Dey *et al.*, 2012; Ma *et al.*, 2013).

Another approach for achieving genotype independence is to identify specific genes associated with tissue culture responses. Quantitative trait loci (QTL) mapping studies to identify genomic regions associated with callus induction and plant regeneration have been carried out in grasses such as barley, maize, and wheat (Amer *et al.*, 1997; Mano and Komatsuda, 2002; Salvo *et al.*, 2018). Although these studies concluded that tissue culture response is a complex polygenic trait, further investigation of specific candidate genes is needed, especially in sorghum, to identify genetic mechanisms that control somatic embryogenesis and efficient regeneration response.

### Improved transformation efficiency

Successful introduction of a wide range of genes of interest into sorghum will depend on efficient tissue culture and transformation protocols. Currently, sorghum transformation typically uses indirect somatic embryogenesis, which goes through the callus stage. The maintenance of callus cultures is labor intensive and a lengthy process that can induce somaclonal variation. Direct somatic embryogenesis is an alternative that has been achieved in maize and sugarcane (Taparia *et al.*, 2012; Lowe *et al.*, 2018), and could be applied to sorghum to shorten tissue culture time and increase efficiency. As shown in maize, introduction of morphogenic regulators enables immature embryos to transition into somatic embryos in a few days and allows bypassing the callus stage (Lowe *et al.*, 2018). Alternatively, the tissue culture method using leaf whorls reported by Silva *et al.* (2020) could be adapted to induce direct somatic embryogenesis as previously demonstrated in sugarcane (Desai *et al.*, 2004; Taparia *et al.*, 2012).

A promising alternative to somatic embryos is using embryogenic cell suspension cultures, which have been used to transform switchgrass, with high efficiency of 85% (Ondzighi-Assoume *et al.*, 2019), and cotton, reaching transformation efficiency of ~19% (Ke *et al.*, 2012). Efficient methods for maintaining sorghum cell cultures have potential to improve transformation efficiency by reducing somaclonal variation, decreasing false positives, and increasing the survival rate of transgenics. Additionally, cells with a synchronized cell cycle could be obtained, which may benefit CRISPR/Cas genome editing studies aiming for homology-directed repair (HDR). Cells have different abilities to repair double-stranded breaks using the non-homologous end joining (NHEJ) or HDR pathways, and the phase of the cell cycle plays a major role in the choice of the pathway (Heyer *et al.*, 2010). The HDR pathway activity is restricted to the late S and G_2_ phases of the cell cycle, while NHEJ occurs during the entire cell cycle . Therefore, cell suspension cultures can be a valuable tool not only to improve transformation efficiency, but also to increase genome editing efficiencies for targeting gene insertions, replacements, or stacking.

Other approaches to improve transformation efficiency involve the development of more efficient DNA delivery methods. Although progress has been made in *Agrobacterium*-mediated transformation, engineering strains with increased virulence and a wider host range will be necessary to boost efficiency. Optimizations to avoid overgrowth of *Agrobacterium* in the tissue culture selection media will also be relevant (Ahmed *et al.*, 2018). Another promising strategy is the use of nanoparticles to deliver DNA, which has already been demonstrated in wheat and cotton leaves, resulting in strong protein expression (Demirer *et al.*, 2019).

### Genome editing

CRISPR/Cas-mediated genome editing can be applied broadly, including creating mutant collections of specific genes that have not been well characterized, creating variations for breeding purposes, and altering regulatory elements. CRISPR/Cas9-mediated gene editing in sorghum was first reported using *Agrobacterium*-mediated transformation to restore the function of an out-of-frame red fluorescence protein (DsRED2) through NHEJ (Jiang *et al.*, 2013). Since then, CRISPR/Cas9 delivery by *Agrobacterium* has been adopted to mutate several sorghum genes (A. Li *et al.*, 2018; Che *et al.*, 2018; Char *et al.*, 2020). To date, only one protocol for CRISPR/Cas9 genome editing using particle bombardment has been published (G. Liu *et al.*, 2019).

Cas9 requires a 5ʹ-NGG-3ʹ protospacer adjacent motif (PAM) site upstream of the sgRNA-binding region in the genome. Other endonucleases, such as Cpf1 that targets T-rich regions (Zetsche *et al.*, 2015), have not yet been exploited in sorghum. These alternative endonucleases broaden the range of sequences that can be targeted. In cases where the goal is generating precise point mutations, an alternative to the low-efficiency HDR pathway is using the CRISPR base editors (Komor *et al.*, 2016). CRISPR base editors allow cytosine to thymine or adenine to guanine base editing, and have been widely adopted to introduce targeted substitutions in other crops such as rice and wheat to improve important agricultural traits, such as flowering time and herbicide resistance (C. Li *et al.*, 2018; Kang *et al.*, 2018; Zhang *et al.*, 2019; Li *et al.*, 2020).

### Prevention of transgene flow

Valid concerns about transgene flow to sorghum’s sexually compatible wild weedy relatives have dampened commercial interest in engineered cultivars. Therefore, techniques that prevent transgene introgression or propagation through pollen should be prioritized. Alternatively, transgene-free methods for genome editing such as the delivery of a pre-assembled ribonucleoprotein (RNP) complex, which is done via protoplast transfection or particle bombardment, can be used (Woo *et al.*, 2015; Svitashev *et al.*, 2016; Liang *et al.*, 2017). Particle bombardment would be the most suitable method for sorghum as it does not require plant regeneration from protoplasts, an ongoing challenge for sorghum tissue culture. Distinct methods adopted in other species also have potential in sorghum. For example, Zhang *et al.* (2016) generated transgene-free and homozygous wheat mutants in the T_1_ generation by transiently expressing *Cas9* in callus cells.

Another promising strategy to impede transgene flow into the wild would be the delivery of transgenes into chloroplasts to take advantage of their maternal inheritance. This avoids transgene transmission via pollen, closing a potential escape route into the environment (Daniell, 2002). Thus, chloroplast transformation would allow stable introduction of *Cas9* into sorghum’s chloroplast genome to generate Cas9 lines that would not propagate the transgene via pollen.

## Current genetic, genomic, and bioinformatic resources

Sorghum has several characteristics that make it an excellent potential model species for grass research. It is a diploid (2*n*=20), which makes it more amenable to genetic and genomic studies compared with polyploid bioenergy crops such as sugarcane. It also has a small genome size (~730 Mbp) compared with maize (2.5 Gbp), sugarcane (~10 Gbp), and wheat (~17 Gbp) (Paterson *et al.*, 2009). Extensive variations across cultivated and wild species have been identified, suggesting a rich genetic source for adaptation and engineering (Tao *et al.*, 2021). Additionally, sorghum is a C_4_ grass with high nitrogen and water use efficiency (Ghannoum *et al.*, 2011) and complements other grass models such as rice and *Brachypodium*, which are C_3_ grasses. The wide genetic variation found within and among sorghum cultivars is also attractive as it can be exploited to improve the crop through breeding, population genetic, and quantitative genetic approaches (Satish *et al.*, 2016). To support the adoption of a plant species as a research system, it is critical to have accessible resources, including germplasm collections, reference genome sequences with good quality functional annotations, and easy-to-use informatics tools that collate existing data. While sorghum does have some of these resources, there are still many gaps.

### Genetic resources

The largest sorghum germplasm collection is maintained by the USDA Agricultural Research Service (ARS) National Plant Germplasm System and consists of >40 000 accessions from 114 countries, of which many regional specific subsets have been genetically characterized (Cuevas *et al.*, 2017, 2018; Olatoye *et al.*, 2018; Cuevas and Prom, 2020; Faye *et al.*, 2021). The International Crops Research Institute for the Semi-Arid Tropics (ICRISAT) in India also has a large collection of 37 904 accessions (Morris *et al.*, 2013; Cuevas *et al.*, 2017). A third collection with >16 000 accessions is kept by the National Crop Genebank of China. Information and sources of seeds can be identified via databases such as USDA-ARS GRIN (https://npgsweb.ars-grin.gov/gringlobal/search.aspx), Eurisco (http://eurisco.ecpgr.org/), and Genesys (https://www.genesys-pgr.org/). Additional collections with particular relevance to the use of sorghum as a biomass crop include the biomass association panel (Brenton *et al.*, 2016) and the nested association mapping population (Bouchet *et al.*, 2017). These collections contain immense genetic diversity, which is essential for breeding programs that aim to develop cultivars better adapted to different conditions worldwide and also an important resource to elucidate molecular machineries that lead to traits of interest.

Furthermore, alleles not found in nature can be generated through mutagenesis (e.g. genotoxic chemicals or γ-irradiation) (Xin *et al.*, 2008; Jiao *et al.*, 2016; Chen *et al.*, 2019) or, more recently, through genome editing. Mutant lines are being added to these germplasm collections to create an even more diverse community resource. Increasingly, these mutant populations are accompanied by whole-genome sequences, allowing researchers to take a reverse genetics approach to identifying gene function (Addo-Quaye *et al.*, 2018).

### Genomic resources

The first sorghum reference genome (from the grain sorghum BTx623) was generated using whole-genome shotgun sequencing in 2009 (Paterson *et al.*, 2009), and placed ~98% of the genes in their chromosomal context. More recently, BTx623 version 3.1.1 was released with improved assembly and annotation (McCormick *et al.*, 2018). The high-quality reference genome of the sweet sorghum ‘Rio’ was also recently released using Pacific Biosciences long-read sequencing (Cooper *et al.*, 2019). The authors used it to explore the possible genomic differences between sorghum types, and revealed a high rate of non-synonymous and potential loss-of-function mutations in sweet sorghum. However, few changes in gene content and overall genome structure were observed (Cooper *et al.*, 2019). Two additional genomes, BTx642 and RTx430, are also available on Phytozome (see below). An ongoing sorghum pan-genome project at the DOE Joint Genome Institute (JGI) will explore this information further (Mockler, 2016).

### Bioinformatic resources

Several bioinformatic resources host sorghum data (links and references described in Table 4). Sorghum breeders and researchers can rely on bioinformatic resources such as Phytozome, the Plant Comparative Genomics portal of the DOE Joint Genome Institute (JGI) (Goodstein *et al.*, 2012). This includes the latest sorghum reference genome (McCormick *et al.*, 2018). Additionally, the Sorghum genome SNP database (SorGSD), a database with 62 million single nucleotide polymorphisms (SNPs) from 48 sorghum accessions, allows the user to search for synonymous and non-synonymous SNPs, their annotation, geographic origin, and breeding information (Luo *et al.*, 2016). A valuable resource for sorghum improvement is the Sorghum Genomics Functional Gene Discovery Platform, which enables the identification of sorghum lines containing natural and chemical-induced variations in coding sequences (https://www.purdue.edu/sorghumgenomics/)(REF). The Sorghum Functional Genomics Database (SorghumFDB) also has a search feature with orthologous pairs in *A. thaliana*, rice, and maize, in addition to gene family classifications, gene annotations, loci conversions, miRNA and target gene information, and a genome browser (Tian *et al.*, 2016). The PlantGDB, a resource for comparative plant genomics, has a section on sorghum (SbGDB), which includes gene structure annotation, sequence analysis tools, and annotated protein alignments. Also, sorghum metabolic network data can be found in SorghumbicolorCyc at the Plant Metabolic Network (PMN), a curated source of metabolic information from the literature and computational analyses (Schläpfer *et al.*, 2017). Lastly, UniProt has sorghum protein sequences from genome sequencing projects (Saski *et al.*, 2007; Paterson *et al.*, 2009; Hawkins *et al.*, 2021). These resources can assist researchers who are new to sorghum research to understand sorghum genome architecture and its variations, and to draw comparisons with other extensively studied species.

**Table 4.** Bioinformatics resources available for sorghum research

Bioinformatic tool	Purpose	Website	Source	Reference
Phytozome	Reference genome and alignment searches	https://phytozome.jgi.doe.gov/pz/portal.html#!info?alias=Org_Sbicolor	Joint Genome Institute (JGI)	McCormick *et al.*, (2018)
Plant Metabolic Network	Network of metabolic pathway data	https://plantcyc.org/content/sorghumbicolorcyc-7.0.1	Carnegie Institution for Science	Hawkins *et al.*, (2021)
Gramene sorghum	All sorghum resources as statistics, germplasm resources, metabolic pathways	https://archive.gramene.org/species/sorghum/sorghum_intro.html	Cold Spring Harbor Laboratory and Cornell University	Tello-Ruiz *et al.*, (2018)
Sorghum FDB - Functional Genomics Database	Integrated search for gene family classifications, gene annotations, miRNA and target gene information, orthologous pairs in Arabidopsis, rice, and maize, gene loci conversions and a genome browser	http://structuralbiology.cau.edu.cn/sorghum	Zhen Su’s group at China Agricultural University	Tian *et al.*, (2016)
SbGDB	Sequence-centered genome view with focus on gene structure annotation	http://www.plantgdb.org/SbGDB/	Brendel group at Indiana University	–
Uniprot	Proteomic data	https://www.uniprot.org/proteomes/UP000000768	UniProt Consortium	–
Sorghum genomics - Functional Gene Discovery Platform	Search for lines containing natural and *ems*-induced variations in coding sequences	https://www.purdue.edu/sorghumgenomics#	Purdue University	–

## Conclusion

Sorghum has a bright future as a multipurpose crop that is suited to the challenging growth conditions that climate change will bring. Its extensive genetic diversity combined with relatively recent and limited domestication means that it also has an excellent potential for further improvement. Sorghum can become a model system for other grass species, particularly in areas such as abiotic and biotic stress responses, plant–microbiome interactions, and evolution. We see transformation challenges as a major bottleneck to the development of sorghum as both a widely adopted research system and a key feedstock for the bioeconomy, and contend that research tackling this problem is a high priority.
